# Benign Pigmented Dermal Basal Cell Tumor in a Namibian Cheetah (*Acinonyx jubatus*)

**DOI:** 10.1155/2016/7981765

**Published:** 2016-09-26

**Authors:** Sonja K. Heinrich, Bettina Wachter, Gudrun Wibbelt

**Affiliations:** ^1^Leibniz Institute for Zoo and Wildlife Research, Department Evolutionary Ecology, Alfred-Kowalke-Strasse 17, 10315 Berlin, Germany; ^2^Leibniz Institute for Zoo and Wildlife Research, Department Wildlife Diseases, Alfred-Kowalke-Strasse 17, 10315 Berlin, Germany

## Abstract

A 3.5-year-old wild born cheetah (*Acinonyx jubatus*), living in a large enclosure on a private Namibian farm, developed a large exophytic nodular neoplasm in its skin at the height of the left shoulder blade. We describe the clinical appearance, the surgical removal, and histological examination of the tumor, which was diagnosed as a moderately pigmented benign basal cell tumor. A three-year follow-up showed no evidence of recurrence after the surgery. Although neoplasia is reported in nondomestic felids, only very few concern cheetahs. So far, no case of basal cell tumor was described in this species.

## 1. Introduction

The number of reports on neoplasms in exotic felids is continuously rising, particularly due to an increase in longevity in captive animals [1]. Several types of neoplasia are published in captive cheetahs, but no basal cell tumor has been described so far [2–6]. Basal cell tumors arise from nonkeratinizing cells that originate in the basal layer of the epidermis and are one of the most common skin tumors in domestic cats [7].

Namibia hosts the worldwide largest free-ranging cheetah population with most of these animals roaming on privately owned commercial farmland [8, 9]. While some farmers regularly eliminate cheetahs to reduce the threat to their livestock and game animals [8], others keep cheetahs in large enclosures on their guest farms as tourist attractions. Such privately kept animals have to go through an annual health check by an authorized veterinarian. The cheetah research project of the Leibniz Institute for Zoo and Wildlife Research has been working on Namibian farmland since 2002 and has examined more than 400 free-ranging as well as more than 100 captive cheetahs. In 2011, we removed a benign basal cell tumor from a wild born captive cheetah living on a Namibian farm. This was the first neoplastic lesion found by the project in about 14 years of cheetah research.

## 2. Case Presentation

**Figure 1:**
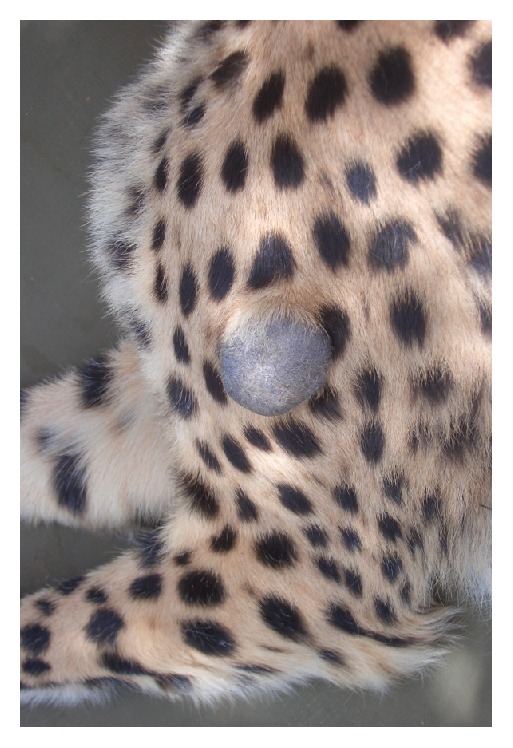
Exophytic solid basal cell tumor of a Namibian cheetah located on the left shoulder.

We were contacted by a local farmer who kept a cheetah on his farm, which exhibited a slow growing alopecic dermal mass at the height of the left shoulder blade that began to grow approximately 1.5 years earlier (Figure 1). The animal was a castrated 3.5-year-old wild born male cheetah, living since the age of 3 months in a large fenced area surrounding the farmhouse. He had been vaccinated yearly against rabies (Rabisin®), feline calicivirus, feline panleukemia virus, feline viral rhinotracheitis virus (Feligen CRP®, Virbac), and feline leukemia virus (Tricat, Nobivac). The surgical option was made. A fine needle aspirate of the mass was not attempted before surgery, as this would have required an additional anesthesia. During the clinical investigation, the cheetah was found in a good health status with a body weight of 47 kg.

The cheetah was immobilized with a mixture of ketamine (3.2 mg/kg, Ketavet®, Kyron Laboratories, Benrose, RSA) and medetomidine (0.06 mg/kg, Novartis, Spartan, Republic of South Africa) administered with a dart shot from a dartgun (Telinject, Dudenhofen, Germany). The skin around the mass was shaved and the surgical field was washed and disinfected with 70% ethanol. The mass and covering skin were excised from the adjacent tissue. The neoplasia was well encapsulated and no infiltrative growth into the surrounding tissue was visible, but it had one large supportive blood vessel, which was ligated and cut. The wound was closed with a subcutaneous consecutive suture and several cutaneous suture stiches both with absorbable material. The animal was treated prophylactically with 2 mg/kg ketoprofen (Ketofen®, Merial) as anti-inflammatory and pain medication and with a combination of 15.000 IU/kg procaine benzyl penicillin and 15.000 IU/kg benzathine benzyl penicillin (Peni-LA Phenix®, Virbac) to prevent a wound infection. To keep the wound clean from dust and dirt, we dressed the animal with children's shirt, which was removed by the farmer seven days after surgery. The immobilization was reversed with Atipamezole (0.11 mg/kg, Antisedan®, Pfizer, RSA). The wound remained uninfected and was nearly invisible at the annual medical check-up one year after the surgery and the cheetah was still alive 3.5 years after removal of the tumor without any signs of recurrence.

The excised neoplasia was fixed in 10% buffered formalin and shipped to Germany in full compliance with the Convention on International Trade in Endangered Species (CITES) for histological examination at the Leibniz Institute for Zoo and Wildlife Research in Berlin.

**Figure 2:**
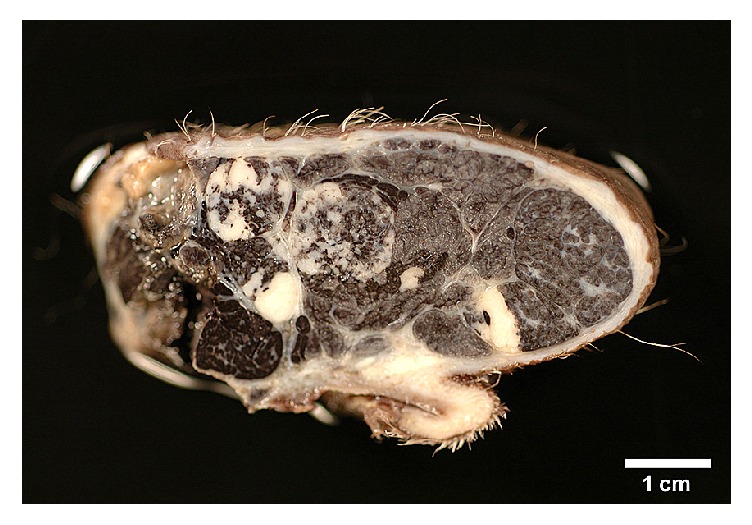
Cut surface of the solid basal cell tumor in a Namibian cheetah (5.5 cm in diameter).

**Figure 3:**
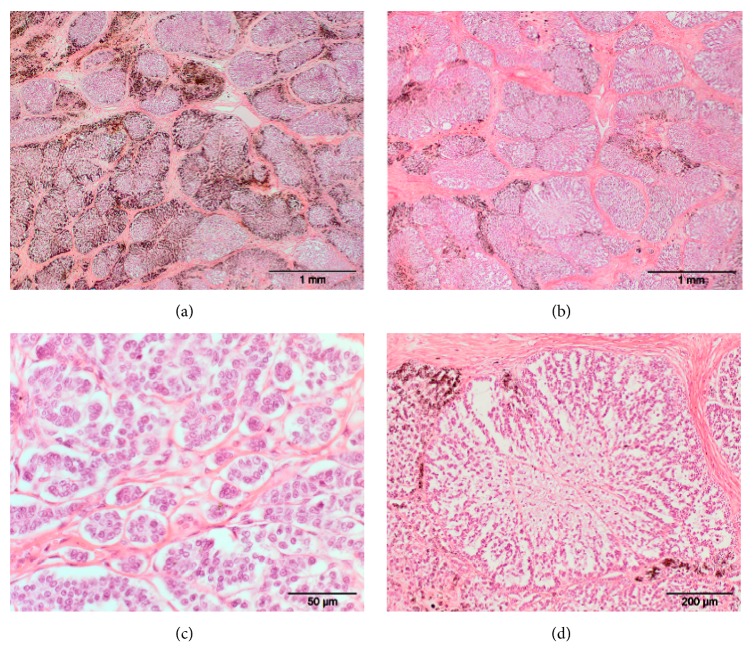
Basal cell tumor in a Namibian cheetah. (a) Area of heavily pigmented dark brown tumor lobules and (b) tumor lobules sparsely pigmented. (c) Tumor cells oriented in cords and nests separated by thin collagen. (d) Tumor nodule with cells with indistinct cell borders delineated by fine connective tissue. HE stain.

The tumor presented itself as an exophytic firm encapsulated broad based nodular dermal to subcutaneous mass measuring 5.5 cm × 4.5 cm × 2.5 cm, covered by intact sparsely haired dark pigmented skin. Cut surfaces revealed multiple small lobules separated by fine white strands of connective tissue (Figure 2). Lobules were mottled light to dark grey or whitish with multinodular solid growth pattern and they did not exceed the surgical excision margins. Microscopically, some lobules were populated by high numbers of heavily dark brown, pigmented cells, while others contained lightly pigmented cells, which were mostly distributed in the periphery of each lobule (Figures 3(a) and 3(b)). Within the lobules, cells were oriented in cords and trabeculae or small islands separated by thin collagen, while distinct sheets of collagen-rich connective tissue surrounded the entire lobules (Figure 3(c)). Rarely, small assemblies of dark pigmented cells (melanophages) were found in the separating connective tissue septa. The neoplastic cells were cuboidal to polygonal with uniformly large round to ovoid central nuclei with stippled chromatin and 1-2 nucleoli (Figure 3(d)). Cytoplasm was scant to moderate, pale eosinophilic, sparsely stained and finely granular. The majority of the tumor cells had indistinct cell borders (Figures 3(c) and 3(d)). Occasionally, single mitotic figures were found (one per 10 high power fields). The overlying epidermis was made of five to six even layers of unremarkable keratinocytes containing some melanosomes in the basal cells as well as some cells of the stratum germinativum. There was mild orthokeratosis and a few remaining hair follicles. The superficial dermis contained mild nodular perivascular neutrophilic infiltrates and dermal collagen was markedly thickened as a response to the expanding tumor, but no other pathological changes were apparent.

A panel of immunohistochemistry stains was applied and revealed tumor cells being positive for pancytokeratin but negative for MelanA, S100, and vimentin. Although the dark pigmentation and nests and islands of polygonal cells prompted the differential diagnosis of a melanocytoma, the immunohistochemistry results lead to the final diagnosis of a moderately pigmented basal cell tumor.

## 3. Discussion

Basal cell tumors are common cutaneous epithelial neoplasms in domestic dogs and cats [10] originating from cells of the basal layer of the epidermis but without epidermal or adnexal differentiation [11, 12]. Two distinct growth forms, cystic and solid basal cell tumors, are found with the former representing the majority of cases in cats [7]. Their microscopic appearance is notoriously pleomorphic with many pattern variations [13], but some features are commonly described such as the dermal position or the singularity in occurrence of these nodular masses. In solid basal cell tumors the cell morphology often varies, whereas the nuclei are usually uniformly ovoid and surrounded by scant cytoplasm [13]. While this holds true for the solid tumor described here in the Namibian cheetah, the majority of its lobules featured a slightly different cellular growth pattern than expected for a basal cell tumor. The nests and clusters of neoplastic cells as well as the sometimes high amount of dark brown pigmentation lead to the first assumption of a melanocytoma. However, basal cell tumors often also contain pigment [14]. One survey on basal cell tumors in cats described 25% of 46 tumors as black or grey masses [10], while another review of 56 of cystic and solid feline basal cell tumors in cats found almost 50% pigmented [7]. Thus, confusion between melanocytoma and basal cell tumors is a recognized obstacle [15]. The application of immunohistochemistry stains including epithelial as well as melanocytic markers [16, 17] was necessary to lead to the diagnosis of a basal cell tumor. An additional differential diagnosis is the trichoblastoma. This benign neoplasia is derived from the trichoblastic epithelium, the primitive hair germ of embryonic follicular development, and occurs in four different variants: ribbon, trabecular, granular, and spindle cell trichoblastoma [18]. Their distinct growth pattern differentiates these entities from the tumor described here, as they usually comprise one to two layers of cells oriented in long and narrow winding cords of cells as well as adnexal differentiation. The trabecular trichoblastoma would appear as the closest resemblance of the tumor in the cheetah. But the characteristic peripheral palisading growth of trabecular trichoblastoma cells is not a feature in the tumor of the animal described here. Currently, there are ongoing discussions whether basal cell tumors should still be recognized as an own entity or whether they should be integrated into trichoblastomas together with all neoplasms deriving from cells of the hair follicle [19, 20]. But until this debate is resolved, we will use the term basal cell tumor.

The most common location of this neoplasia in domestic cats is the head or the trunk [11], while the tumor found in the cheetah was removed at the height of the left shoulder blade. Basal cell tumors are considered benign neoplasms which is also reflected in this case because, although the tumor was of rather large size, three years after removal no sign of recurrence on the skin of the cheetah was visible.

In nondomestic felids, a similar solid basal cell tumor was described in a captive Indian leopard (*Panthera pardus fusca*) [21] and a basal cell epithelioma was found in a captive African lion (*P. leo*) [22]. However, also malignant dermal melanomas have been described in exotic cat species. One was detected in a lion, located at the upper lip and successfully treated with a combination of radio- and immunotherapy [23], and the other was reported in a white tiger (*P. tigris tigris*), which had metastasized to most lymph nodes and the lung [24].

In cheetahs, the most common neoplasia is myelolipoma, which was first described in 1968 [5]. It occurs only in captive cheetahs and is reported with an unusually high frequency [4, 25–27]. It is mostly located in the liver and spleen and it has been suggested as an indicator for chronic disease or stress [26]. Other reports on neoplasia in captive cheetahs include three cases of fibroleiomyoma [3, 4], a mesothelioma [28], and a T-cell lymphoma associated with feline leukemia virus [2]. This is the first description of a solid basal cell tumor in a cheetah. After approximately 14 years of cheetah research in Namibia and no other cases published in the literature, it seems that the described neoplasm occurs, unlike to domestic cats, very rarely in this animal species.
